# The ancestral environment of teosinte populations shapes their root microbiome

**DOI:** 10.1186/s40793-024-00606-0

**Published:** 2024-08-29

**Authors:** Christopher J. Barnes, Maria Sophie Bünner, M. Rosario Ramírez-Flores, Ida Broman Nielsen, Jazmin Ramos-Madrigal, Daria Zharikova, Chloee M. McLaughlin, M. Thomas Gilbert, Ruairidh J.H. Sawers

**Affiliations:** 1 Department of Agroecology, Faculty of Technical Sciences, Aarhus University, Forsøgsvej 1, Slagelse, 4200 Denmark; 2 Centre for Evolutionary Hologenomics, The Globe Institute, Faculty of Health, University of Copenhagen, Copenhagen, Denmark; 3 Departamento de Biotecnología y Bioquímica, Centro de Investigación y de Estudios Avanzados (CINVESTAV-IPN), Irapuato, Guanajuato, 36821 México; 4 Bioscience Division, Oak Ridge National Laboratory, 1 Bethel Valley Rd, Oak Ridge, TN 37830 USA; 5 Department of Plant Science, The Pennsylvania State University, State College, University Park, PA USA; 6 University Museum, NTNU, Trondheim, Norway; 7 The Globe Institute, Øster Voldgade 5 -7, Copenhagen K, 1350 Denmark

**Keywords:** Microbiome, Teosinte, Maize, Plant-microbiome coevolution, Hologenome, Holobiont, Adaptation

## Abstract

**Background:**

The composition of the root microbiome affects the host’s growth, with variation in the host genome associated with microbiome variation. However, it is not known whether this intra-specific variation of root microbiomes is a consequence of plants performing targeted manipulations of them to adapt to their local environment or varying passively with other traits. To explore the relationship between the genome, environment and microbiome, we sampled seeds from teosinte populations across its native range in Mexico. We then grew teosinte accessions alongside two modern maize lines in a common garden experiment. Metabarcoding was performed using universal bacterial and fungal primers to profile their root microbiomes.

**Results:**

The root microbiome varied between the two modern maize lines and the teosinte accessions. We further found that variation of the teosinte genome, the ancestral environment (temperature/elevation) and root microbiome were all correlated. Multiple microbial groups significantly varied in relative abundance with temperature/elevation, with an increased abundance of bacteria associated with cold tolerance found in teosinte accessions taken from high elevations.

**Conclusions:**

Our results suggest that variation in the root microbiome is pre-conditioned by the genome for the local environment (i.e. non-random). Ultimately, these claims would be strengthened by confirming that these differences in the root microbiome impact host phenotype, for example, by confirming that the root microbiomes of high-elevation teosinte populations enhance cold tolerance.

**Supplementary Information:**

The online version contains supplementary material available at 10.1186/s40793-024-00606-0.

## Background

Microbes are present on nearly every surface of plants where they play an important functional role for their hosts [56]. The root microbiome is the most diverse site of plant-microbe interactions, and its composition has been shown to have far-reaching effects [4]. For example, differences in the root microbiome have been linked to differences in crop yields [59] and the chemical defences in the leaves (Sharma, Anand and Kapoor, [55]). Therefore the composition of the root microbiome affects the host’s growth (Berendsen, Pieterse and Bakker, [11]). There has been a large number of studies investigating how the environment influences the root microbiome, with soil pH [5], soil nutrients [25] and climate [6, 37], all influential. Differences have been observed in the root microbiome between plant species [26], and there is also interest in understanding within species variation of the root microbiome [14, 42]. Differences in the root microbiome between genotypes have been found repeatedly; direct correlations between the root microbiome and the phylogenetic relatedness of the hosts have yielded inconclusive results (i.e. whether more closely related plants also have more similar microbiomes) [14, 19, 42, 48]. Differences in the root microbiome associated with genome-wide variation (within species) are generally smaller than that of environmental variation [42]. However, intra-specific variation in the root microbiome may contribute to differences in the phenotype, such as growth rates [39] and resilience to abiotic stresses [9, 52]. If this can be established, it would pave the way for crop microbiomes to be manipulated through breeding programmes (or directly through genetic modification) to provide additional growth benefits (Ravanbakhsh, Kowalchuk and Jousset, [51]). However, the relationship between the genome and microbiome is confounded by environmental variation [19]. Thus there is a need for large-scale systematic studies to disentangle the complex relationship between the host genome, microbiome and the environment before microbiome engineering can become a reality [3].

As maize is one of the most widely grown crops globally [22], its root microbial community has been subjected to extensive investigation. While results of such studies have revealed that maize’s root microbiome composition differs between lines [14], with increased abundances of specific microbes linked to various traits, such as drought tolerance [9], it remains inconclusive whether such intra-specific differences are (i) deterministically encoded for within the genome, and (ii) contribute meaningfully to differences in host functioning, such as tolerance to abiotic stresses. A major challenge to performing microbiome studies on maize, and indeed other common crop species, is that the domestication process has likely warped the relationship between the host and its microbiome. These crops have been bred with exogenous water and fertiliser application, which may have undermined their ability to utilise microbes to mitigate abiotic stresses (such as drought) and maximise nutrient use efficiency. Under favourable growing conditions, plants invest fewer resources (in the form of rhizoexudates) into their root microbiome [21]. Thus there may have even been active selection against supporting a diverse and robust root microbiome by breeders to minimise root exudates in favour of higher yields [46]. Meanwhile, wild crop relatives have evolved with their local soil microbes in less favourable growing conditions and in competition with other plant species. Therefore, there is a selection pressure on them to better utilise microbes to improve their tolerance to abiotic stresses and nutrient resource efficiency. Studies attempting to link host genome variation, environmental variation and the root microbiome have predominantly been performed using domesticated crop species [14, 19, 48]. However, the genomes of modern crops are regulated by both natural selection and domestication processes, which almost certainly confounds its relationship to the microbiome and environment, which is avoided by studying wild plant species.

Teosinte is the progenitor of modern-day maize. It grows naturally within a highly heterogeneous environment, where it is adapted to elevation and temperature gradients across Mexico [2]. Two sub-species that are endemic to Mexico have been identified, the lowland *Zea mays* subsp. *Parviglumis* from which modern maize lines were originally derived, and a highland form, *Z. mays* subsp. *Mexicana* [29] that has been demonstrated as the source of considerable post-domestication introgressions into maize lines [7]. It is known that the teosinte root microbiome differs from modern maize [15], however the root microbiomes of *Parviglumis* and *Mexicana* have not been compared. While the root microbiome of teosinte has had time to co-evolve over evolutionary timescales for the local environmental conditions, the selection pressures for modern maize are very different, and likely shaped by the domestication process. Studying teosinte plants therefore allows for the root microbiome to be investigated from natural populations that have known systematic genome variation (different sub-species) that are known to be adapted to different environments (lowland and highland regions of Mexico). Taking seeds from these teosinte accessions and growing them within controlled environments would allow for the effects of genome variation on the root microbiome to be quantified, without confounding environmental variation [3]. Further, correlations between the host genome, environment and the root microbiome would add considerable weight to the claim that the root microbiome is manipulated by the host for specific functions (i.e. adaptation to local environmental conditions) and are not just random differences between genotypes.

In this project, our aim was to investigate whether the root microbiomes of teosinte accessions differed from their ancestral environment when grown in a common garden experiment. Seeds were taken from teosinte populations that span their ancestral range in Mexico, which spans over a 2,500 m elevational gradient and grown in a common garden experiment. Additionally, modern lines (B73 and CML312) were grown alongside teosinte plants as comparisons. After 12 weeks of growth, roots were sampled, and the root bacteria and fungi were independently profiled using universal primers for each. We then assessed whether (a) both the modern inbred lines individually fell inside the natural variation of teosinte or had their own unique root microbial assembly and (b) whether the ancestral environment and/or genome variation of wild teosinte accessions are systematically associated with variation in the root microbiome.

## Methods

### Experimental overview

**Fig. 1:**
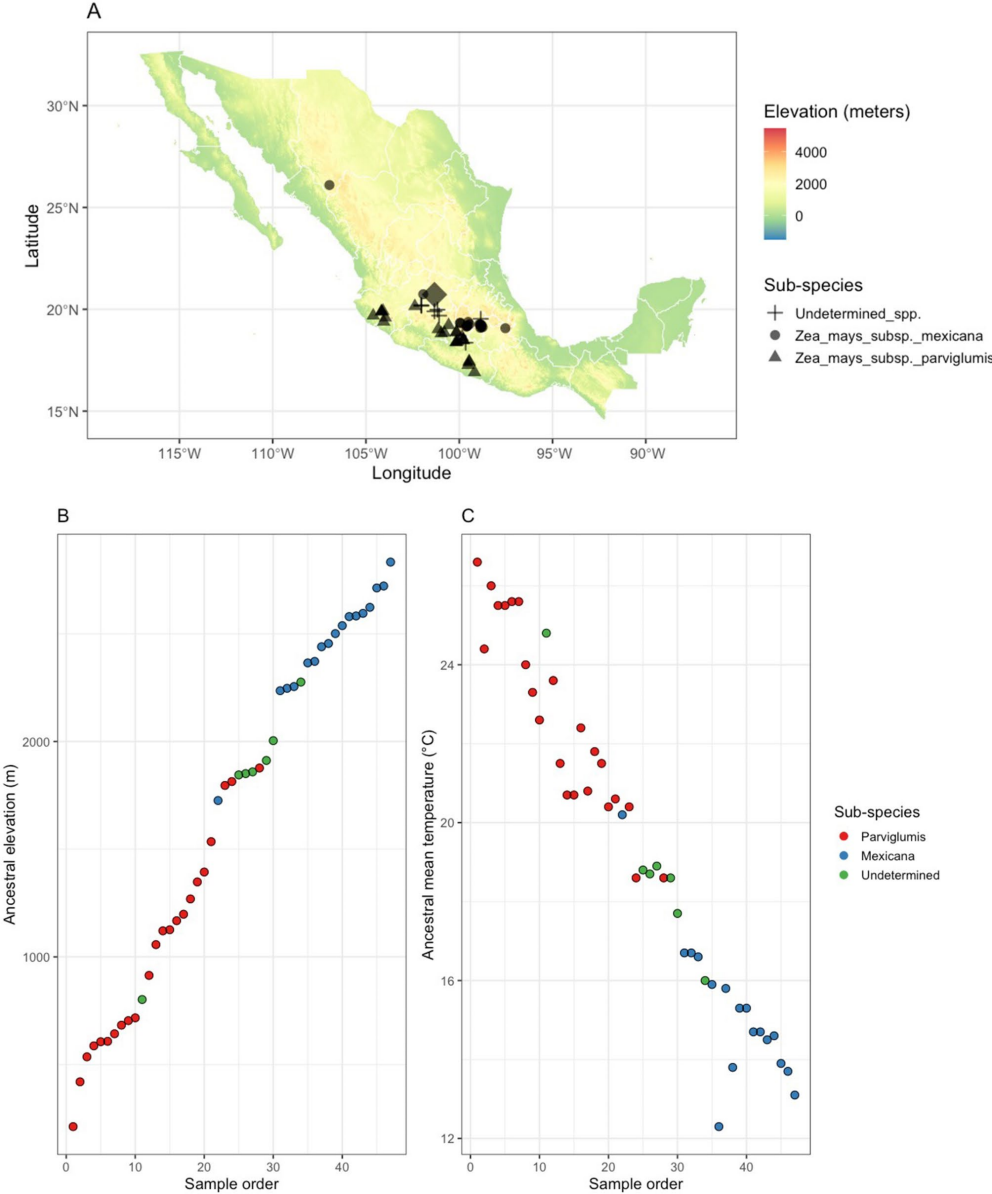
(**A**) Map of sample collections. In total, 47 accessions of teosinte were sampled from across Mexico (illustrated as crosses). These came from two sub-species, spanning a considerable (**B**) elevational and (**C**) temperature gradients. These were grown alongside two modern lines in a common garden experiment (at the diamond) and their root microbiomes compared

This work was performed as a common garden experiment in Irapuato, Guanajuato, Mexico (20.720189°, -101.328720°) at 1,724 m in elevation, during the summer of 2017, with roots sampled from mature plants. In total, the roots of 9 and 10 different plants from the modern lines CML312 and B73 were sampled respectively. Additionally, the roots from single teosinte plants were sampled from 47 accessions of teosinte (a mixture of *Zea mays subsp. mexicana* and *Zea mays subsp. parviglumis*) that originated from a range of geographical locations and elevations but were grown together here. This included 23 *Parviglumis* samples (lowland), 17 *Mexicana* samples (highland) and 7 samples that were likely hybrids (found at intermediate elevations) (Fig. 1). 

Teosinte plants are unlikely to grow if directly planted in the field. Therefore, seeds from teosinte accessions and modern maize plants were germinated in sheet pots (0.5 L) under controlled greenhouse conditions, consisting of an average temperature of 24 °C and humidity of 48%. These sheet pots were filled with pre-wetted substrate (consisting of homogenised soil taken from the common garden), with three seeds per pot added after scarification with overnight soaking. Teosinte and maize seedlings were thinned to one plant after germination. After approximately 30 days seedlings were transplanted to the common garden (Irapuato, Guanajuato, Mexico) as whole soil plugs to minimise disruption to the root networks.

The plot consisted of furrows (rows), with one furrow comprised of only CML312 plants, followed by a second comprised of only B73 plants. Accessions were then randomly planted in a snaked structure across further furrows (no accession could be adjacent to each other). Furrows were 24 m long, and plants were planted with 0.5 m between them along the furrows, and 0.7 m between plants of adjacent furrows. Furrows had a minimum of 2.5 m distance to the edge to avoid edge effects and were surrounded predominantly by modern maize lines.

Due to time and resource limitations, one plant of each teosinte accession was sampled, which was performed 12 weeks after transplanting into the field, corresponding to approximately their flowering time. Meanwhile, repeated sampling of the CML312 (9 plants) and B73 (10 plants) lines across the entire row was performed to gauge any bias along the rows. Since modern NILs were not interspersed with the teosinte accessions, there was a small risk that spatial variation drives differences between the two maize NILs, and between NILs and teosinte root microbiomes. However, the larger distances along the furrows (24 m) was taken into account within the experimental design compared to the smaller distances between furrows (0.7 m) that was not. Further, differences in the root microbiome between modern maize lines and teosintes have also been shown to be relatively large [15]; Favela, Bohn and Kent, [23]; Huang et al., [36, 42, 58].

### Sampling, root processing, and DNA extraction

Teosinte/maize roots were dug up ~ 20 cm away from the stalk and up to 20 cm in depth. Fine nodal roots (approximately under 2 mm in diameter) were randomly collected from the upper 20 cm of the soil profile and placed into 50 mL Falcon tubes. For the collection of bulk soil samples, samples were collected from between rows 3 and 4 approximately 1 one third from the top and bottom edges. This was performed using a hand trowel and digging until the end of the blade (20 cm) was level with the ground and repeating to extract a 5 cm x 5 cm core. Root and bulk soil samples were stored at -80 °C until the following process. Before the DNA extraction, the roots were vigorously washed and sonicated with sterile phosphate-buffer saline solution (PBS; 1.37 M NaCl, 27 mM KCl, 100 mM Na_2_HPO_4_, and 18 mM KH_2_PO_4_) at 7.5 pH. Roots were placed in a new tube and were rinsed with sterile distilled water several times. After washing, roots were dried with paper towels and then frozen in liquid nitrogen to grind root samples using a Tissuelyzer II (QIAGEN) for 1 min at 30 Hz. DNA extraction was then performed in a dedicated pre-PCR laboratory within the Globe Institute of the University of Copenhagen. Initially, 100 mg of freeze-dried roots, and 250 mg of freeze-dried bulk soil, was weighed out into 1.5 mL plastic tubes. These samples underwent DNA extraction as per the manufacturer’s protocol of the MoBio PowerSoil DNA Isolation kit (MoBio Laboratories, Solana Beach, CA, USA) Fig. 1.

### Sequencing preparation

For bacterial data, metabarcoding was performed as per Lund et al. [42]. Briefly, PCRs were performed using the 341F and 806R primers which target the v3-v4 region of the 16S bacterial gene. Fungi were analysed as per Frøslev et al. [27]. Briefly, PCR was performed using the gITS7 and ITS4, which target the ITS2 region. Both sets of primers had 8–9 bp internal tags within to multiplex samples. Each sample was run using three separate primer combinations which served as technical replicates. The bacterial libraries were prepared using a Blunt-End-Single-Tube (BEST) approach [18] that was specifically modified for metabarcoding, while the fungal libraries were built using a TruSeq PCR Free Kit (Illumina, CA, US). For each, four library pools were created and run on two separate sequencing runs performed using an Illumina MiSeq v3 platform (250 bp paired-ends). Extraction and PCR negatives were run in triplicate (three of each negative type) alongside the bacterial and fungal primer sets.

### Environmental data

We collected data that summarised the environment of origin for each teosinte individual. Climate data was extracted from the WorldClim database [24]. Monthly and annual average potential evapotranspiration (PET), and a measure of aridity (mean annual precipitation divided by mean annual PET) were collected from the CGIAR-CSI Globality-Arbitdty database [61]. Information on inter-annual variability in precipitation was calculated with data from the NCEP/NCAR Reanalysis project [38]. Inter-annual variability in precipitation was obtained by calculating each calendar month’s coefficient of variation (CV) across years for each month’s surface precipitation rate.

Edaphic chemical and physical properties were collected from SoilGrids [35] and the Global Soil Dataset (GSD) [54]. Data from GSD includes soil features of the topsoil and 1 m below the surface. We found high concordance of values for topsoil and 1 m below the surface and excluded the topsoil data from our dataset. All soil variables were cleaned by removing outliers and imputed missing values using the MICE package (Buuren and Groothuis-Oudshoorn, [16]).

### Data processing

Bacterial data was analysed as in Lund et al. [42] using DADA2. Briefly, DADA2 was used to filter, predict error rates and correct them, combined ends and remove chimeras. DADA2 aggregates reads into amplicon sequence variants (ASVs), which represent error corrected exact sequences that can delineate closely related bacterial taxa [17]. ASVs were then annotated using DADA2’s native RDPClassifier algorithm against the Silva138 database. Here, ASVs not annotated to the bacterial kingdom were removed, and so too were reads assigned to chloroplast and mitochondria. The fungal data was processed as per Frøslev et al. [28]. Here fungal data was processed exactly as the bacteria until ASV formation. ASV sequences underwent extraction using ITSx to extract only the ITS2 region. These ITS2 sequences then underwent clustering into operational taxonomic units (OTUs) using a 98.5% clustering threshold, whichbetter represents fungal species, and was deemed adequate for addressing biodiversity questions [13] while avoiding inflating diversity with ITS repeats from the same fungus. Finally, OTU sequences were assigned taxonomy using the UNITE fungal general release database with dynamic OTU clustering (28/10/2022 release) using BLAST. OTUs not assigned to the fungal kingdom were removed. For both datasets, technical replicates were combined by filtering out ASVs/OTUs that were detected in single samples, and then reads from replicates were pooled into single samples. At this point, the extraction and PCR negative control samples were left with nominal amounts of reads for both bacteria and fungi (> 100 reads), suggesting low rate of contamination and no longer considered. For the bacteria, many reads were assigned to the chloroplast and removed, leaving 517,657 reads from an initial 17,441,683 reads, accounting for a mean of 7,613 reads per sample. For the fungi, a total of 3,961,348 from a total of 16,984,327 reads were retained, accounting for a mean of 55,965 reads per sample. Raw read counts then underwent a fourth root transformation and converted to relative abundances for use in downstream compositional analyses. For estimates of phylogenetic diversity, a RaxML tree was created using Geneious Prime software (v2003.2.1; for bacteria and fungi separately) and calculated using the relative abundance data using the *Vegan* package of *R* [47].

### Phylogenetic analyses of teosinte accessions

Genomic data was available for 39 of our teosinte samples (the 7 undetermined, 18 *Parviglumis* samples and 14 *Mexicana*), and therefore samples without genomic data were omitted from downstream phylogenetic analyses (but retained in all other analyses) (Fig. S1). Genome data consisted of allele frequency data present in the Seeds of Discovery database (Hearne, Franco and Chen, [34]), which we used to estimate the genetic distance between pairs of teosinte accessions. This public database consists of allele frequency data generated through pool-sequencing of many of the wild and domesticated maize accessions present in the CIMMYT repository. Data was downloaded from the CIMMYT repository for the relevant accessions. Allele frequencies were used to estimate the FST [12] between pairs of accessions and to build a genomic distances matrix.

### Statistical analyses

Initially, differences in Shannon’s diversity between teosinte accessions, modern lines (analysed as separate groups) and soil were calculated using a Kruskal-Wallis test followed by a post-hoc Dunn Test. Compositional differences were calculated using PERMANOVA and a post-hoc analysis performed using the pairwiseAdonis function (adjusting for multiple comparisons).

Teosinte samples were subsetted from the modern lines and soil samples and compared to environmental parameters, with subsequent statistics performed on the combined teosinte dataset, but also repeated on the individual sub-species datasets (*Parviglumis* and *Mexicana -* excluding hybrids). Numeric metadata parameters underwent z-normalisation before principal component analyses were performed on the soil and climatic data sets independently to simplify data. Three PCs were used for soil and two for climatic variables which explained over 80% of cumulative variation. Additionally, a principal co-ordinates of neighbourhood matrix (PCNM) was constructed using the GPS co-ordinates of ancestral teosinte accessions (using the *geodist* package). PCNM1 and 2 were used in downstream statistics. Finally, elevation (z-normalised) was included as a predictor variable since sampling was performed over mountainous regions of Mexico. For analyses of alpha-diversity, Shannon’s diversity was correlated against individual explanatory parameters using spearman’s rank coefficients. For community composition analyses, forward selection was manually performed by performing individual PERMANOVAs (between community and environmental parameters) and adding parameters with the largest effects sizes to a combined model, until the additional variables were no longer significant. Additionally, correlations between families and individual taxa (relative abundance) and elevation were performed using Spearman’s rank correlation coefficient. The possible interaction between genome and microbiome was analysed using two approaches. A matrix approach was taken, whereby a Bray-Curtis similarity matrix for the bacterial/fungal community was correlated against the phylogenetic distance matrix using Mantel statistics and Procrustes. A second approach involved performing a principal coordinate analysis on the phylogenetic distance matrix, and using the Phylo PCs (1–4) as explanatory variables within PERMANOVA tests. While it is also possible to assess for different evolutionary processes by correlating the phylogeny to the microbiome (with Blomberg’s K and Pagel’s lambda), low percentages of the microbiome were summarised by PCAs of the microbiome, and much lower than the phylogeny, therefore the PCs of the phylogeny were performed instead.

## Results

### The root microbiome of modern maize lines differs from the range of teosinte accessions

**Fig. 2:**
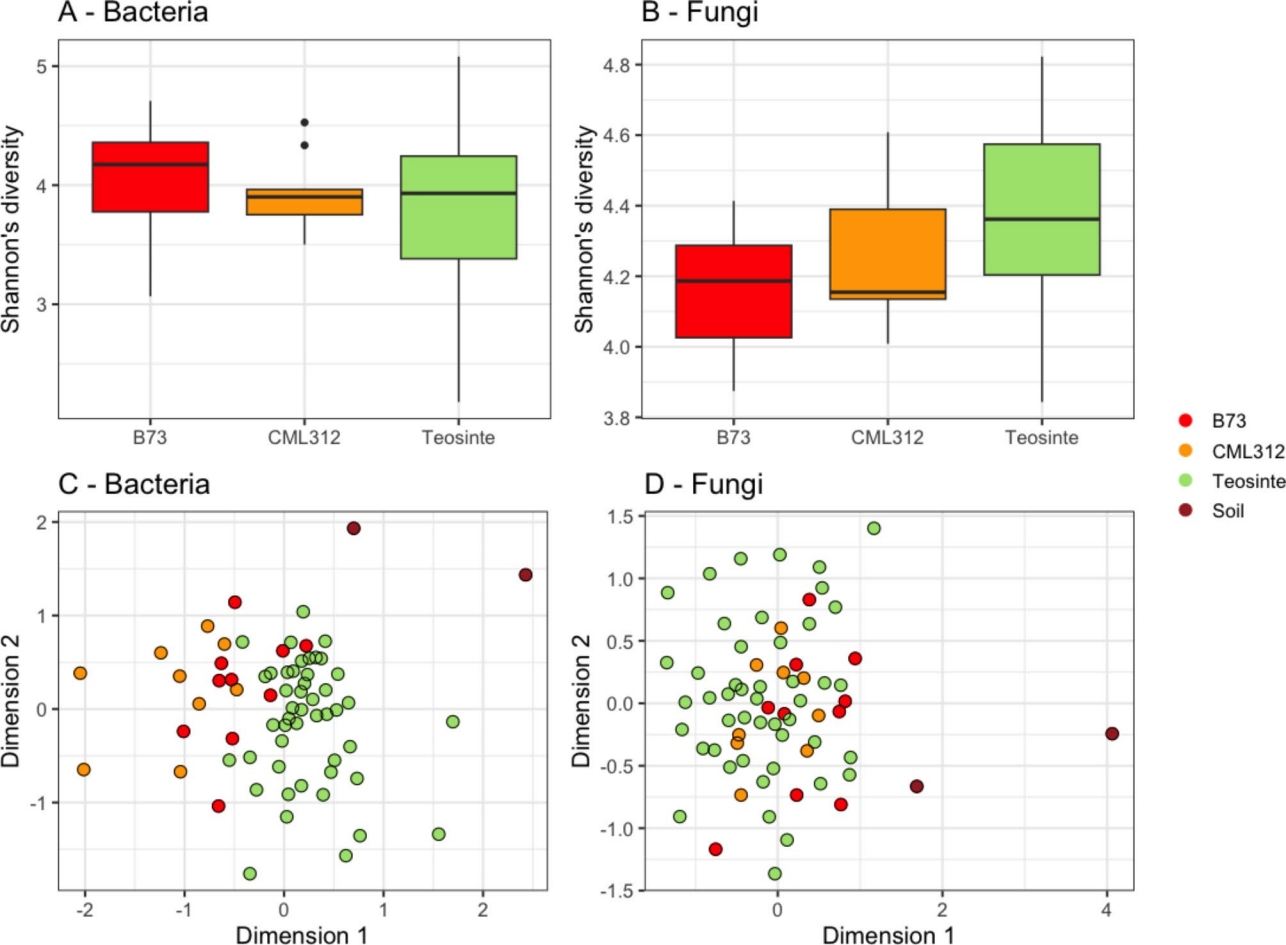
Differences in bacterial (**A**) and fungal (**B**) Shannon’s diversity were visualised in boxplots, finding limited differences between sample types. Bars represent the lower and upper quartiles, and the line represents the median. Whiskers represent the end of observed data points, except for the dots, which represent outliers (defined as greater than 1.5 times the inter quarter range). Soil Shannon’s diversity from soil was 3.2 and 4.3 for bacteria, and 3.8 and 4.0 for fungi. However, the bacterial (**C**) and fungal (**D**) composition each differed between each sample type (i.e. maize lines differed from each other, teosinte accessions and soil), which were visualised using non-metric multidimensional scaling

Initially, we tested whether the root microbiome of domesticated lines (B73 and CML312) was outside of the natural variation of teosinte. Shannon’s diversity was compared between samples. For bacteria, there were no significant differences in Shannon’s diversity found between the modern lines, teosinte accessions and soil (*H* = 1.99, *P* = 0.575; Table S1; Fig. 2A). However, for the fungi, there were significant differences between samples (Fig. 2B) (*H* = 11.15, *P* = 0.011), being the highest in the teosinte accessions (4.37 ± 0.30), followed by CML312 (4.29 ± 0.18), B73 (4.16 ± 0.18) and soil had the least (3.88 ± 0.15) fungal Shannon’s diversity. A post-hoc Dunn test revealed no significant differences between individual comparisons (Table S1).

There were more significant differences between sample types when comparing community compositions. For bacteria, an initial PERMANOVA revealed significant differences between sample types (*R*^2^ = 0.110, *P* = 0.001), and post-hoc analysis revealed all sample types differed from each other (Table S1; Fig. 2C). Similarly, the composition of fungi was found to be significantly different between samples using PERMANOVA (*R*^2^ = 0.083, *P* = 0.001). Post-hoc analyses revealed significant differences between all root communities (B73, CML312 and teosinte accessions), but neither modern line significantly differed from soil (Table S1). Interestingly, a greater percentage of fungal variation was explained by sample type compared to the bacteria, and they also showed more distinct clustering in the ordinations (Fig. 2D).

### The root microbiome of teosinte correlates with their parents’ environment

As we have teosinte originating from different environments, we were next able to explore whether the teosinte root microbiome varied with their ancestral environment. The parental environment was then correlated with their progenies root microbiome from the common garden experiment. For both the bacteria and fungi, the community composition correlated with ancestral environment in the forward selection models (Table S2), accounting for ~ 4% of variation in each of the bacterial and fungal communities (Bacteria - *R*^2^ = 0.045, *P* = 0.003; Fungi - *R*^2^ = 0.039, *P* = 0.002). However, there were also correlations between ancestral elevation, climate and original sampling geographic location (PCNM1), and climate (PC1) correlated with the community composition within individual community analyses (but did not correlate after elevation was added in the forward selection models) (Table S2).

Shannon’s diversity was also explored in relation to the parent’s environment. Here we found that only PCNM1 (ancestral geographic location) correlated with bacterial Shannon’s diversity (*R*_s_=0.42, *P* = 0.004) and no environmental parameters correlated with fungal Shannon’s diversity (Table S2).

### The temperature-elevation-geographic gradient was correlated with many individual taxa

**Fig. 3:**
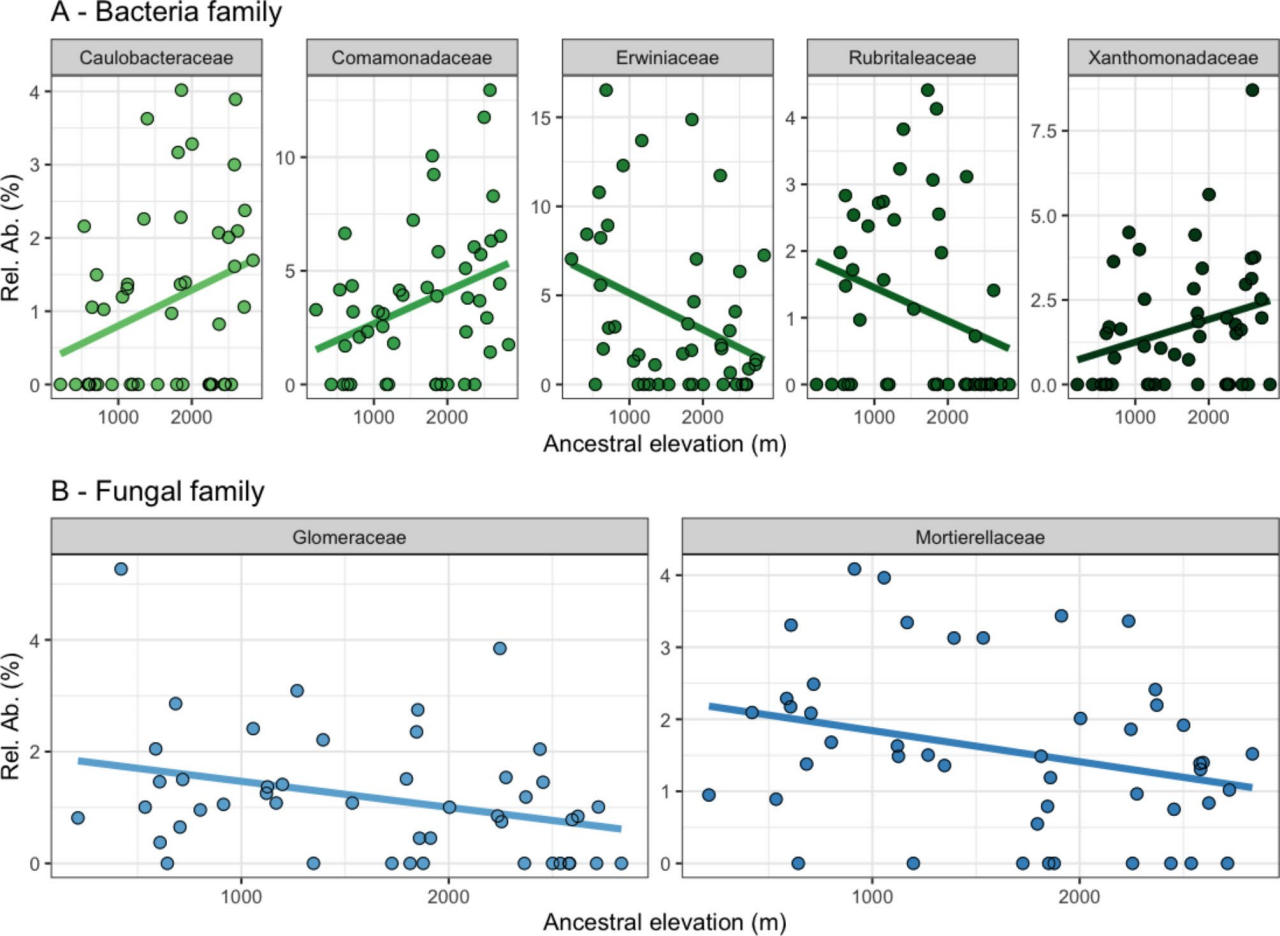
The relevant abundances of abundant families (present in 5 or more samples) were correlated against ancestral elevation revealing many potential correlations between both the bacteria and fungi (significance at *P*-levels but not after correction for multiple comparisons). Note for visualisation purposes only, untransformed elevation was plotted (by z-normalised within statistics)

Elevation (of the parent accession) was used in statistical analyses, but as a representative for itself, climate and geography. This was performed as elevation explained the most variation in the community analyses, and it remains stable unlike the climatic variables. Initially, correlations between ancestral elevation and individual family relative abundances were performed (Table S3). There were five bacterial families that varied with elevation (*P*-values). However, due to low sample numbers and a high number of families (resulting in many tests), these variations were not significant when *P*-values were adjusted for multiple comparisons (FDR; *q*-values). This included the Erwiniaceae which declined in relative abundance in the roots of samples whose parents came from higher/colder temperatures (*R*_s_=-0.340, *P* = 0.021, *q* = 0.158) (Fig. 3A), while the Comamonadaceae also showed the inverse relationship (*R*_s_=0.310, *P* = 0.035, *q* = 0.177; Table S3). The Caulobacteraceae (*R*^2^ = 0.334, *P* = 0.021, *q* = 0.158) and Xanthomonadaceae (*R*_s_=0.292, *P* = 0.046, *q* = 0.175) increased in relative abundance with ancestral elevation, being almost absent in the accessions originating from sea level and rising to approximately 2% mean relative abundance at 3,000 m elevation. Meanwhile, the Rubritaleaceae showed almost the exact inverse relationship (*R*_s_=-0.332, *P* = 0.023, *q* = 0.158), falling from approximately 2% in accessions originating from sea level to absence in accessions taken at high elevations (3,000 m; Table S3).

For the fungi, there were fewer trends between relative abundance and ancestral elevation (Fig. 3B), with two families varying with elevation at the *P*-value level, and none were significant after being adjusted for multiple comparisons (*q*-values; Table S3). This included the Glomeraceae (*R*_s_=-0.353, *P* = 0.015, *q* = 0.315), which were shown to decline with ancestral elevation from approximately 1.8% at sea level to 0.2% at 3,000 m in elevation. Additionally, the Mortierellaceae declined with ancestral elevation from approximately 2% at ancestral sea levels to 1% at 3,000 m (*R*_s_=-0.290, *P* = 0.048, *q* = 0.333).

**Fig. 4:**
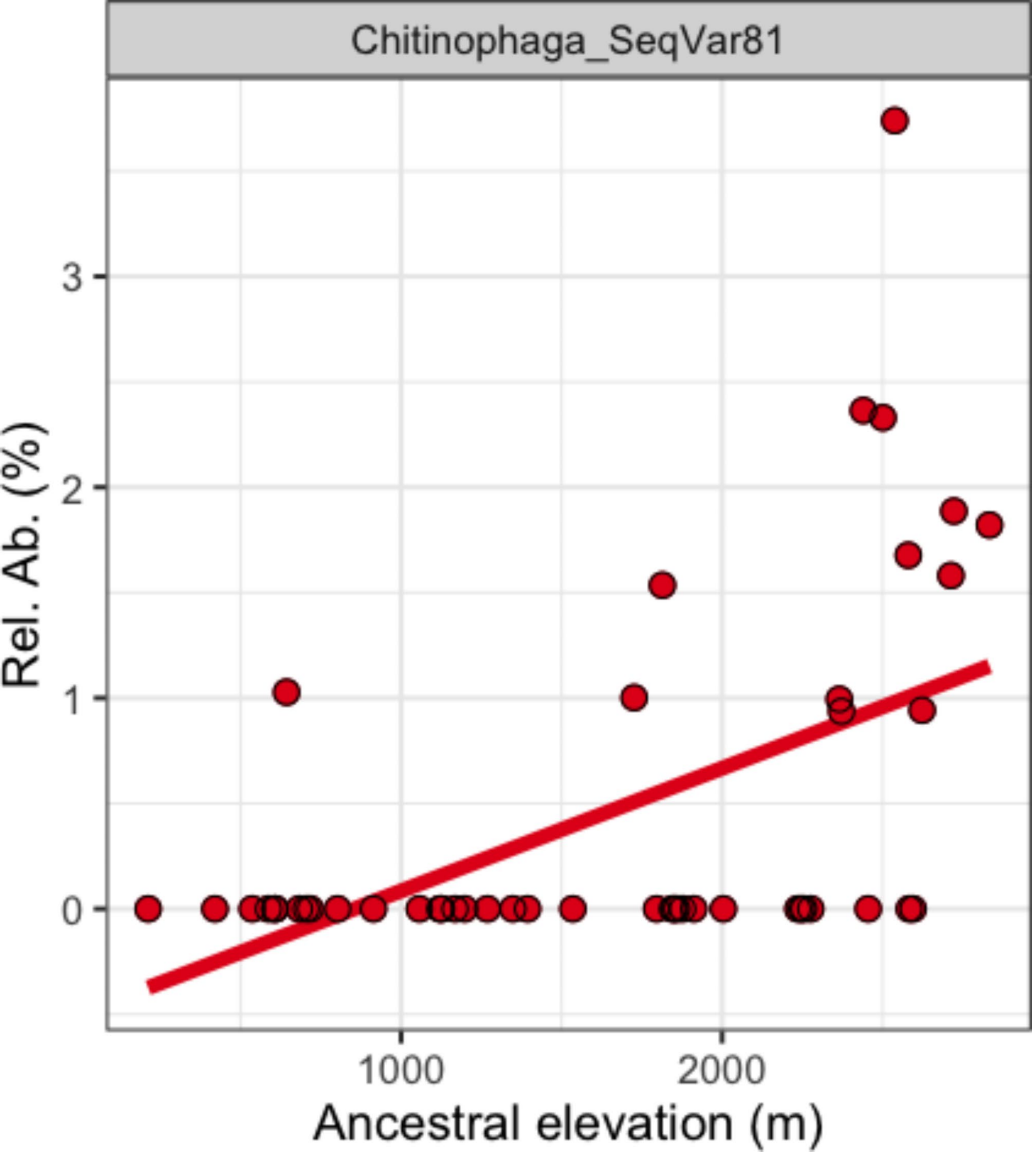
The relative abundances of abundant taxa were correlated against ancestral elevation revealing a *Chitinophaga ginsengisoli* (ASV81) that positively increased in abundance with ancestral elevation after adjusting for multiple comparisons. Note for visualisation purposes only, untransformed ancestral elevation was plotted (by z-normalised relative abundance was used within statistics)

We additionally searched for individual taxa that significantly changed in relative abundance with ancestral elevation (Table S4). There was one bacterial ASV assigned to *Chitinophaga ginsengisoli* (ASV81) that significantly increased with ancestral elevation (*R*_s_=0.550, *P* = 0.001, *q* = 0.010), which was absent in the accessions taken from sea level and rose to approximately 1% of the total community in accessions taken at high elevations (3,000 m; Fig. 4). There were however a further 33 ASVs that significantly varied in the unadjusted *P*-values but not after correction for multiple comparisons (Fig. S2**A**).

As in the family analyses, analyses of individual fungal OTUs showed fewer significant patterns of variation with elevation than the bacteria, with no OTUs correlating within *q*-values, and 23 OTUs correlating using *P*-values (Fig. S2**B**; Table S4). These were from a range of different families, but dominated by the Ascomycota phylum, including multiple potential pathogens. There were two OTUs assigned to *Fusarium oxysporum* that were correlated with elevation (at *P*-value only), with one significantly increasing, while the other decreasing (Table S4). There were further OTUs assigned to a *Phoma* species (OTU360), *Bipolaris sorokiniana* (OTU372) and *Exserohilum turcicum* (OTU328) that were putative pathogens that increased in relative abundance with ancestral elevation.

### The evolutionary history of teosinte accessions correlated with the root microbiome

A phylogenetic distance matrix was calculated from genome data and used to correlate the host genetic similarity against the root bacterial and fungal communities (Bray-Curtis matrices) (for the 39 out of 49 teosinte samples that had genome data available). Here, the bacterial community did not correlate with phylogeny using both Mantel (*R* = 0.074, *P* = 0.191) and Procrustes (*R* = 0.745, *P* = 0.142) analyses. However, the fungal community did for both Mantel (*R* = 0.248, *P* = 0.001) and Procrustes (*R* = 0.824, *P* = 0.002) statistics (Table 1). Since these statistics greatly simplify the phylogenetic variation, principal coordinate analysis was also performed on the phylogenetic distance matrix, and the resulting principal coordinates (phylo-PCs) were used as explanatory variables within PERMANOVAs for the microbial communities (Table S5). Within these PERMANOVAs, the Phylo-PC1 correlated with both the bacteria and fungi, accounting for approximately 4% of community variation in each (Bacteria - *R*^2^ = 0.045, *P* = 0.003; Fungi - *R*^2^ = 0.039, *P* = 0.002). Additionally, Phylo-PC4 correlated with the fungal community, accounting for another 4.5% of community variation (*R*^2^ = 0.045, *P* = 0.008). As a supporting analysis, phylogeny was correlated directly against ancestral elevation, explaining 14.7% of phylogenetic variation (using PERMANOVA - *R*^2^ = 0.147, *P* < 0.001).

**Table 1:** Teosinte genome data was used to construct a phylogenetic distance matrix. This was then correlated against the Bray-Curtis similarity matrices for the bacteria and fungi using Mantel and Procrustes statistics

	Mantel statistic		Procrustes permutation	
Comparison	correlation	p-value	correlation	p-value
Phylogenetic and bacterial dataset	0.074	0.191	0.745	0.142
Phylogenetic and fungal dataset	**0.248**	**0.001**	**0.824**	**0.002**

**Fig. 5:**
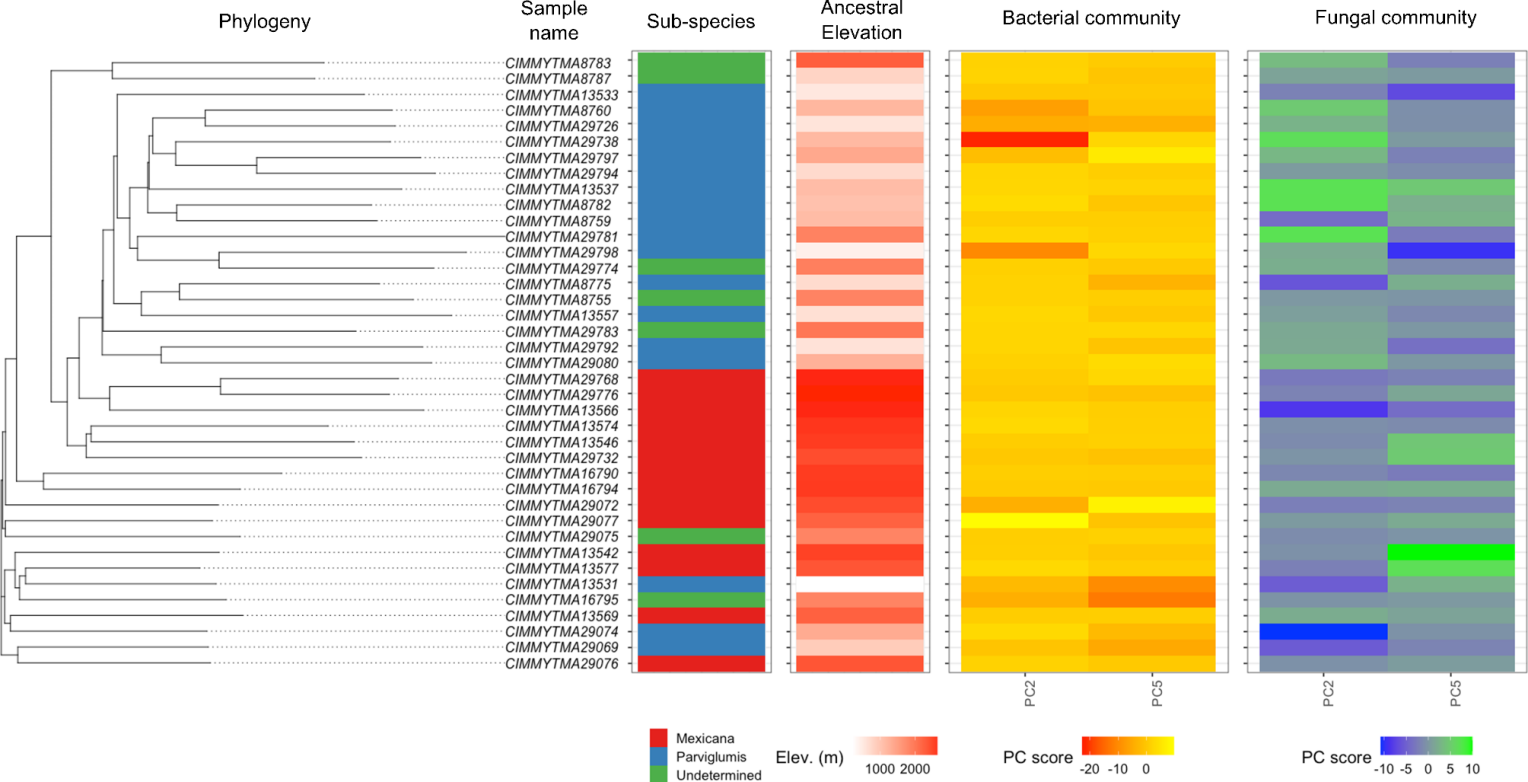
A phylogenetic tree was created using genomic data from 39 teosinte accessions. Here the interplay between the phylogeny was linked to the bacterial (as principal components 2 and 5 [PC2 and PC5]) and fungal (also PC2 and 5) communities, elevation and sub-species using heatmaps

We assigned samples to highland and lowland sub-species using morphology and phylogenetic data and explored for differences in their root microbiomes. In total there were 23 *Parviglumis* samples (lowland), 17 *Mexicana* samples (highland) and 7 that were likely hybrids based on genomic and morphological data. These were samples taken from intermediate elevations, further suggesting they were likely hybrids. Therefore, we excluded the likely hybrid samples and re-analysed for a sub-species effect, finding a significant difference in community between them (Bacteria - *R*^2^ = 0.045, *P* = 0.005; Fungi - *R*^2^ = 0.041, *P* = 0.005) but not in Shannon’s diversity (Bacteria - *W* = 0.042, *P* = 0.837; Fungi - *W* = 0.651, *P* = 0.420). Finally, we reran a PERMANOVA test to correlate ancestral elevation against the microbial communities while using sub-species as a conditional variable (i.e. controlling for it), finding a significant effect of elevation across sub-species (Bacteria - *R*^2^ = 0.052, *P* = 0.002; Fungi - *R*^2^ = 0.047, *P* = 0.006). We therefore explored the differences in the root microbiome associated with ancestral elevation between this sub-species (Fig. 5).

### Patterns of variation with mexicana and Parviglumis

Sub-species were re-analysed individually to explore for associations between the ancestral elevation/temperature variation (i.e. their adapted regions) and their root microbiomes (Table S6). The composition of the root bacteria from *Parviglumis* significantly correlated with climate PC2 (*R*^2^ = 0.089, *P* = 0.004) and ancestral elevation (*R*^2^ = 0.068, *P* = 0.018) within the final model. Bacterial Shannon’s diversity significantly varied with the ancestral climate PC2 (*R*_s_=-0.513, *P* = 0.012), soil PC2 (*R*_s_=0.577, *P* = 0.004), and PCNM1 (*R*_s_=0.449, *P* = 0.032) using *Parviglumis* samples only (Table S6). Meanwhile there were no significant correlations (neither composition nor Shannon’s diversity) between the root fungi of *Parviglumis* plants and any ancestral environmental properties. For the *Mexicana* samples, there were no correlations between the ancestral environment and bacterial composition, but PCNM1 (ancestral geography) negatively correlated with bacterial Shannon’s diversity (*R*_s_=-0.657, *P* = 0.005). However, the fungal communities of *Mexicana* correlated with ancestral elevation, explaining 8.6% of community variation (*R*^2^ = 0.086, *P* = 0.022). There were also no correlations with fungal Shannon’s diversity and the explanatory variables (Table S6).

Correlations between individual families and ancestral elevation were repeated using the divided sub-species datasets (Table S7). In general, the change in both fungal and bacterial abundance was consistent between sub-species, but significance was further limited by low sampling number. For example, the Chitinophagaceae (*Parviglumis*: *R*_s_=0.289, *P* = 0.181 *q* = 0.426; *Mexicana*: *R*_s_=0.056, *P* = 0.831, *q* = 0.873) and Comamonadaceae (*Parviglumis*: *R*_s_=0.430, *P* = 0.041 *q* = 0.285; *Mexicana*: *R*_s_=0.302, *P* = 0.240, *q* = 0.623) increased in abundance with ancestral elevation in both sub-species and the Erwiniaceae decreased in both (*Parviglumis*: *R*_s_=-0.464, *P* = 0.026 *q* = 0.270; *Mexicana*: *R*_s_=-0.305, *P* = 0.233, *q* = 0.622) (Table S7), but none significantly.

Since the *Chitinophagales ginsengisoli* OTU(81) significantly increased in abundance with ancestral elevation in the combined dataset, it was analysed between sub-species. It trended upwards within both *Parviglumis* (*R*_s_=0.346, *P* = 0.001 *q* = 0.814) and *Mexicana* (Table S8), although non-significantly (*R*_s_=0.345, *P* = 0.174, *q* = 0.814). Meanwhile, the other individual taxa were not robustly explored between sub-species as there were simply too few positive occurrences in the low number of samples to proceed reliably with.

Finally, we correlated the phylogeny to the microbial communities of each sub-species. However, there were no significant correlations between the genome and microbiome within neither the matrix-based approaches (Mantel and Procrustes) nor the principal coordinate-based approach (Table S9).

### Insights into the root microbiome of modern maize from teosinte accessions

Since the modern maize lines used in this study were domesticated for colder, temperate regions (as most are), we expected the two modern lines to cultivate more microbes associated with colder temperatures/higher elevation. We therefore compared the relative abundances of the families of the five bacterial and fungal species that correlated most strongly with ancestral elevation (Table S10). For the bacteria, only the Rubritaleaceae significantly differed (*H* = 28.89, *P* < 0.001), with it being markedly higher in the CML312 line compared to all other sample types (Fig. S3A). While not significantly different, the modern lines cultivated equal to or higher abundances of Caulobacteraceae (*H* = 8.15, *P* = 0.008), Comamonadaceae (*H* = 5.53, *P* = 0.237) and Xanthomonadaceae (*H* = 9.02, *P =* 0.060), all of which increased with elevation. For the fungi, only the Pleosporaceae significantly differed between groups (*H* = 10.32, *P =* 0.035; Table S10), being significantly higher in the B73 line than all other sample types, while CML312 and the lowland teosinte accessions had lowest relative abundances (Fig. S3B). The other fungal families showed little difference in relative abundance between the elevations, and between the teosinte accessions and modern lines.

## Discussion

In this work, we shed new information on understanding the regulation of the root microbiome, finding that the ancestral environment correlates with the root microbiome of the progeny. While it has been well documented that there are differences in the root microbiome both between and within plant species [32, 42], there are conflicting results as to whether this variation is random [48], or vary as a consequence of differences in regulation by the plant [14]; Matus-Acuña, Caballero-Flores and Martínez-Romero, [43]; Lund et al., [42]. Here we found that the microbiome and genome (phylogeny) were correlated, suggesting this variation is non-random. Further, we found a correlation between this genome-microbiome pattern and the ancestral environment (elevation/temperature), suggesting differences in the genome pre-condition the root microbiome for their environment. Further, these effects might be obfuscated within modern crops during the domestication process, of which the majority of this type of research has been tested in [14, 53]; Gholizadeh, Mohammadi and Salekdeh, [31]). A strength of this work is that the common garden experiment was performed at an intermediate elevation within teosinte’s natural range, likely exposing them to much of their native soil microbiome which they have potentially co-evolved with [50]. However, one additional consideration is that the teosinte accessions found at the lowest and highest elevations were likely exposed to increasingly dissimilar soil microbiomes, and future experiments would benefit from performing reciprocal transplants (i.e. growing lowland species in highland and vice versa) to confirm that differences in the regulation of the root microbiome are consistently observed despite changing soil microbiomes.

Further support for teosinte accessions being able to manipulate their root microbiome comes from more detailed analyses of these compositional changes. Interestingly, the bacterial families Comamonadaceae, Caulobacteriaceae and Xanthomonadaceae were all found to be enriched in domesticated maize under cold stress [10], and here we found these to be in higher abundance in the plants taken from higher ancestral elevations/lower temperatures (before *P*-adjusting), and in higher abundances in the modern maize lines that have been bred for a temperate environments that are more exposed to cold/chilling stresses. Despite the underlying spatial variation potentially confounding this relationship (the modern lines were not interspersed with the modern lines, but grown adjacently), several of these families have also been found to be enriched in other plant species under cold-stress [30, 49]. Together, these suggest that teosinte plants can shape their root microbiome for specific functions, and cold tolerance in this instance. Microbial symbionts have been proven to be capable of improving their host’s resilience to low temperatures [1], and increases in the abundance of these microbes would clearly benefit the host. While the process of fine-scale manipulating the root microbiome must undoubtedly be complex, multiple mechanisms by which this could be achieved have been identified. For example, changes in root morphology [40], and changes in the composition and rate of rhizoexudates have been shown to affect the composition of the root microbiome (Baudoin, Benizri and Guckert, [8]), with the root morphology of teosinte accessions previously linked to their ancestral environment [44]. While less is known about the rhizoexudates of teosinte, the benzoxazoids are rhizoexudates produced by maize [20] (and other cereal crops [41]) that have been shown to attract and repel specific bacteria [45]. Therefore, this process of root microbiome optimisation is not outside of the realm of possibility. Indeed, a recent study of maize landraces linked a single gene to ancestral soil nitrogen and the root bacteria [33].

For fungi, the Glomeraceae are arbuscular mycorrhizal fungi (AMF) which correlated negatively with ancestral elevation/temperature. Results here are in line with previous findings suggesting that AMF colonisation decreases under cold stress within maize plants (Zhu, Song and Xu, [60]). However, AMF colonisation also provided a greater fitness benefit under cold conditions, emphasising the need to consider the interactions between the host genome, microbiome and environment.

The results were confounded by sampling teosinte accessions from two sub-species associated with low- and highlands [29]. When samples were partitioned into individual sub-species, the power of statistical analyses was limited by low sampling numbers (particularly of *Mexicana*). However, within sub-species, we still observed correlations between ancestral elevation/temperature and the root microbiome (but not between host genomic variation and the root microbiome within sub-species). Further investigations of the bacterial and fungal families suggested that they trended similarly over the ancestral elevation/temperature gradient across sub-species, although again, results were limited in statistical power. It therefore seems likely that results here were not exclusively driven by differences in sub-species. Repeating this experiment with larger sampling numbers, biological replicates and incorporating the temporal-spatial variability of the microbiome into analyses would improve the robustness of these results. Finally, it needs to be confirmed that the observed compositional differences in the root microbiome do impact the host’s cold tolerance in a biologically meaningful way [57].

## Conclusions

Here we found that the host genome, ancestral environment and root microbiome were correlated. This would suggest that plants can, at least partially, influence their root microbiome to promote specific functions. However, we next need to go beyond correlations to determine whether these differences in the composition influence the host phenotype. Given the importance of microbes to many plant functions [56], and the number of mechanisms which plants can influence root microbes (Baudoin, Benizri and Guckert, [8]; King et al., [40], we strongly believe they do. Ultimately, if this can be comprehensively proven, it would confirm that the root microbiome can be manipulated as a trait within breeding programmes to promote the growth and resilience of crops (Ravanbakhsh, Kowalchuk and Jousset, [51]).

### Electronic supplementary material

Below is the link to the electronic supplementary material.

Supplementary Material 1

Supplementary Material 2

## Data Availability

Raw demultiplexed sequencing reads are available on the NCBI’s sequence read archive (SRA) under the submission (SUB14164647)

## References

[1] Acuña-Rodríguez IS et al. Functional roles of microbial symbionts in plant cold tolerance. Ecology Letters. 2020;23(6):1034–1048. 10.1111/ele.13502 10.1111/ele.13502 32281227

[2] Aguirre-Liguori JA et al. Divergence with gene flow is driven by local adaptation to temperature and soil phosphorus concentration in teosinte subspecies (Zea mays parviglumis and Zea mays mexicana). Molecular Ecology. 2019;28(11):2814–2830. 10.1111/mec.15098 10.1111/mec.15098 30980686

[3] Alberdi A et al. Disentangling host–microbiota complexity through hologenomics. Nature Reviews Genetics. 2021;1–17. 10.1038/s41576-021-00421-0 10.1038/s41576-021-00421-0 34675394

[4] Badri DV, Vivanco JM. Regulation and function of root exudates’, Plant, Cell Environment. 2009;32(6): 666–681. 10.1111/j.1365-3040.2009.01926.x 10.1111/j.1365-3040.2008.01926.x 19143988

[5] Barnes CJ et al. Temporally Variable Geographical Distance Effects Contribute to the Assembly of Root-Associated Fungal Communities. Frontiers in Microbiology. 2016;7. 10.3389/fmicb.2016.00195 10.3389/fmicb.2016.00195 PMC4766365 26941720

[6] Barnes CJ et al. Extreme rainfall affects assembly of the root-associated fungal community, New Phytologist. 2018;220(4):1172–1184. 10.1111/nph.14990 10.1111/nph.14990 PMC6282977 29350759

[7] Barnes AC et al. An adaptive teosinte mexicana introgression modulates phosphatidylcholine levels and is associated with maize flowering time. Proceedings of the National Academy of Sciences, 2022;119(27): e2100036119. 10.1073/pnas.2100036119 10.1073/pnas.2100036119 PMC9271162 35771940

[8] Baudoin E, Benizri E, Guckert A. Impact of artificial root exudates on the bacterial community structure in bulk soil and maize rhizosphere. Soil Biology and Biochemistry. 2003;35(9): 1183–1192. 10.1016/S0038-0717(03)00179-2

[9] Begum N et al. (2019) ‘Improved Drought Tolerance by AMF Inoculation in Maize (Zea mays) Involves Physiological and Biochemical Implications’, Plants, 8(12), p. 579. 10.3390/plants8120579 10.3390/plants8120579 PMC6963921 31817760

[10] Beirinckx S et al. (2020) ‘Tapping into the maize root microbiome to identify bacteria that promote growth under chilling conditions’, Microbiome, 8, p. 54. 10.1186/s40168-020-00833-w 10.1186/s40168-020-00833-w PMC7166315 32305066

[11] Berendsen RL, Pieterse CMJ, Bakker PAHM. The rhizosphere microbiome and plant health. Trends in Plant Science. 2012;17(8):478–486. 10.1016/j.tplants.2012.04.001 10.1016/j.tplants.2012.04.001 22564542

[12] Bhatia G et al. Estimating and interpreting FST: the impact of rare variants. Genome Research. 2013;23(9):1514–1521. 10.1101/gr.154831.113 10.1101/gr.154831.113 PMC3759727 23861382

[13] Blaxter M et al. Defining operational taxonomic units using DNA barcode data. Philosophical Transactions of the Royal Society B: Biological Sciences. 2005;360(1462):1935–1943. 10.1098/rstb.2005.1725 10.1098/rstb.2005.1725 PMC1609233 16214751

[14] Bouffaud M-L et al. Root microbiome relates to plant host evolution in maize and other Poaceae . Environmental Microbiology. 2014;16(9);2804–2814. 10.1111/1462-2920.12442 10.1111/1462-2920.12442 24588973

[15] Brisson VL, et al. Phosphate availability modulates root exudate composition and rhizosphere microbial community in a teosinte and a modern maize cultivar. Phytobiomes J. 2022;6(1):83–94. 10.1094/PBIOMES-06-21-0041-R

[16] Buuren S. van and Groothuis-Oudshoorn, K. mice: Multivariate Imputation by Chained Equations in R’, Journal of Statistical Software. 2011;45:1–67. 10.18637/jss.v045.i03

[17] Callahan BJ et al. DADA2: High-resolution sample inference from Illumina amplicon data. Nature Methods. 2016;13(7):581–583. 10.1038/nmeth.3869 10.1038/nmeth.3869 PMC4927377 27214047

[18] Carøe C et al. Single-tube library preparation for degraded DNA. Methods in Ecology and Evolution, 2018;9(2): 410–419. 10.1111/2041-210X.12871

[19] Chen L et al. Soil Characteristics Overwhelm Cultivar Effects on the Structure and Assembly of Root-Associated Microbiomes of Modern Maize. Pedosphere. 2019;29(3):360–373. 10.1016/S1002-0160(17)60370-9

[20] Cotton TEA et al. Metabolic regulation of the maize rhizobiome by benzoxazinoids. The ISME Journal. 2019;13(7):1647–1658. 10.1038/s41396-019-0375-2 10.1038/s41396-019-0375-2 PMC6592824 30796337

[21] Dungait JAJ et al. (2012) ‘Advances in the understanding of nutrient dynamics and management in UK agriculture’, *Science of The Total Environment*, 434, pp. 39–50. 10.1016/j.scitotenv.2012.04.029 10.1016/j.scitotenv.2012.04.029 22748430

[22] Erenstein O et al. (2022) ‘Global maize production, consumption and trade: trends and R&D implications’, *Food Security*, 14(5), pp. 1295–1319. 10.1007/s12571-022-01288-7

[23] Favela A, Bohn M, Kent A. (2022) ‘N-Cycling Microbiome Recruitment Differences Between Modern and Wild Zea mays’, *Phytobiomes Journal*, 6(2), pp. 151–160. 10.1094/PBIOMES-08-21-0049-R

[24] Fick SE, Hijmans RJ. (2017) ‘WorldClim 2: new 1-km spatial resolution climate surfaces for global land areas’, *International Journal of Climatology*, 37(12), pp. 4302–4315. 10.1002/joc.5086

[25] Finkel OM et al. (2019) ‘The effects of soil phosphorus content on plant microbiota are driven by the plant phosphate starvation response’, *PLOS Biology*, 17(11), p. e3000534. 10.1371/journal.pbio.3000534 10.1371/journal.pbio.3000534 PMC6876890 31721759

[26] Fitzpatrick CR et al. (2018) ‘Assembly and ecological function of the root microbiome across angiosperm plant species’, *Proceedings of the National Academy of Sciences*, 115(6), pp. E1157–E1165. 10.1073/pnas.1717617115 10.1073/pnas.1717617115 PMC5819437 29358405

[27] Frøslev TG et al. (2019) ‘Man against machine: Do fungal fruitbodies and eDNA give similar biodiversity assessments across broad environmental gradients?’, *Biological Conservation*, 233, pp. 201–212. 10.1016/j.biocon.2019.02.038

[28] Frøslev TG et al. (2022) ‘The biodiversity effect of reduced tillage on soil microbiota’, *Ambio*, 51(4), pp. 1022–1033. 10.1007/s13280-021-01611-0 10.1007/s13280-021-01611-0 PMC8847473 34448122

[29] Fukunaga K et al. (2005) ‘Genetic Diversity and Population Structure of Teosinte’, *Genetics*, 169(4), pp. 2241–2254. 10.1534/genetics.104.031393 10.1534/genetics.104.031393 PMC1449573 15687282

[30] Garcia Mendez S et al. (2023) ‘Unravelling the bacterial community composition of Valerianella locusta, a cold tolerant plant’, *Phytobiomes Journal* [Preprint]. 10.1094/PBIOMES-12-22-0106-R

[31] Gholizadeh S, Mohammadi SA, Salekdeh GH. (2022) ‘Changes in root microbiome during wheat evolution’, *BMC Microbiology*, 22(1), p. 64. 10.1186/s12866-022-02467-4 10.1186/s12866-022-02467-4 PMC8881823 35219318

[32] Hamanishi ET et al. (2015) ‘Poplar trees reconfigure the transcriptome and metabolome in response to drought in a genotype- and time-of-day-dependent manner’, *BMC Genomics*, 16(1), p. 329. 10.1186/s12864-015-1535-z 10.1186/s12864-015-1535-z PMC4437445 25895923

[33] He X et al. (2023) ‘Heritable microbiome variation is correlated with source environment in locally adapted maize varieties’, *bioRxiv*, pp. 2023–01. 10.1038/s41477-024-01654-7 38514787

[34] Hearne S, Franco J, Chen J. (2019) ‘2019 release of SNP allele frequency data for maize accessions in the CIMMYT Germplasm Bank maize collection’. Edited by International Maize and Wheat Improvement Center. Translated by Secretaría de Agricultura y Desarrollo Rural (SADER). CIMMYT Research Data & Software Repository Network. https://hdl.handle.net/11529/10548142

[35] Hengl T et al. (2017) ‘SoilGrids250m: Global gridded soil information based on machine learning’, *PLOS ONE*, 12(2), p. e0169748. 10.1371/journal.pone.0169748 10.1371/journal.pone.0169748 PMC5313206 28207752

[36] Huang J et al. (2022) ‘The rhizospheric microbiome becomes more diverse with maize domestication and genetic improvement’, *Journal of Integrative Agriculture*, 21(4), pp. 1188–1202. 10.1016/S2095-3119(21)63633-X

[37] Jansson JK, Hofmockel KS. (2020) ‘Soil microbiomes and climate change’, *Nature Reviews Microbiology*, 18(1), pp. 35–46. 10.1038/s41579-019-0265-7 10.1038/s41579-019-0265-7 31586158

[38] Kalnay E, et al. The NCEP/NCAR 40-year reanalysis project. Renewable energy. Routledge, p. Vol1_146-Vol1_194; 2018.

[39] Katsenios N et al. (2022) ‘Assessment of plant growth promoting bacteria strains on growth, yield and quality of sweet corn’, *Scientific Reports*, 12(1), p. 11598. 10.1038/s41598-022-16044-2 10.1038/s41598-022-16044-2 PMC9270457 35804096

[40] King WL et al. (2021) ‘The hierarchy of root branching order determines bacterial composition, microbial carrying capacity and microbial filtering’, *Communications Biology*, 4(1), pp. 1–9. 10.1038/s42003-021-01988-4 10.1038/s42003-021-01988-4 PMC8055976 33875783

[41] Kudjordjie EN et al. (2019) ‘Maize synthesized benzoxazinoids affect the host associated microbiome’, *Microbiome*, 7(1), p. 59. 10.1186/s40168-019-0677-7 10.1186/s40168-019-0677-7 PMC6460791 30975184

[42] Lund M et al. (2022) *The Rhizosphere Bacterial Communities Differ Among Domesticated Maize Landraces – an Experimental Confirmation*, p. 2021.12.30.474574. 10.1101/2021.12.30.474574

[43] Matus-Acuña V, Caballero-Flores G, Martínez-Romero E. (2021) ‘The influence of maize genotype on the rhizosphere eukaryotic community’, *FEMS Microbiology Ecology*, 97(6), p. fiab066. 10.1093/femsec/fiab066 10.1093/femsec/fiab066 33930111

[44] McLaughlin CM et al. (2023) ‘Evidence that variation in root anatomy contributes to local adaptation in Mexican native maize’. bioRxiv, p. 2023.11.14.567017. 10.1101/2023.11.14.567017 10.1111/eva.13673 PMC10925829 38468714

[45] Niculaes C et al. (2018) ‘Plant Protection by Benzoxazinoids—Recent Insights into Biosynthesis and Function’, *Agronomy*, 8(8), p. 143. 10.3390/agronomy8080143

[46] O’Brien AM et al. (2021) *Strengthened mutualistic adaptation between teosinte and its rhizosphere biota in cold climates*. preprint. Evolutionary Biology. 10.1101/2021.04.20.440703

[47] Oksanen J, et al. The vegan package. Community Ecol Package. 2007;10:631–7.

[48] Peiffer JA et al.Diversity and heritability of the maize rhizosphere microbiome under field conditions. Proceedings of the National Academy of Sciences. 2013;110(16): 6548–6553. 10.1073/pnas.1302837110 10.1073/pnas.1302837110 PMC3631645 23576752

[49] Persyn A et al. Digging into the lettuce cold-specific root microbiome in search of chilling stress tolerance-conferring plant growth-promoting bacteria. Phytobiomes Journal [Preprint] 2022. 10.1094/PBIOMES-07-22-0044-MF

[50] Raaijmakers JM, Kiers ET. () ‘Rewilding plant microbiomes. Science. 2022;378(6620): 599–600. 10.1126/science.abn6350 10.1126/science.abn6350 36356130

[51] Ravanbakhsh M, Kowalchuk GA, Jousset A. Targeted plant hologenome editing for plant trait enhancement. New Phytologist. 2021;229(2): 1067–1077. 10.1111/nph.16867 10.1111/nph.16867 PMC7820966 32772380

[52] Rojas-Tapias D et al. Effect of inoculation with plant growth-promoting bacteria (PGPB) on amelioration of saline stress in maize (Zea mays). Applied Soil Ecology. 2012;61: 264–272. 10.1016/j.apsoil.2012.01.006

[53] Roman-Reyna V et al. Characterization of the Leaf Microbiome from Whole-Genome Sequencing Data of the 3000 Rice Genomes Project. Rice. 2020;13(1):72. 10.1186/s12284-020-00432-1 10.1186/s12284-020-00432-1 PMC7547056 33034758

[54] Shangguan W et al. A global soil data set for earth system modeling. Journal of Advances in Modeling Earth Systems. 2014;6(1):249–263. 10.1002/2013MS000293

[55] Sharma E, Anand G, Kapoor R. Terpenoids in plant and arbuscular mycorrhiza-reinforced defence against herbivorous insects. Annals of Botany. 2017;119(5);791–801. 10.1093/aob/mcw263 10.1093/aob/mcw263 PMC5378189 28087662

[56] Trivedi P et al. Plant–microbiome interactions: from community assembly to plant health. Nature Reviews Microbiology. 2020;18(11):607–621. 10.1038/s41579-020-0412-1 10.1038/s41579-020-0412-1 32788714

[57] Walters WA et al. Large-scale replicated field study of maize rhizosphere identifies heritable microbes. Proceedings of the National Academy of Sciences. 2018;115(28):7368–7373. 10.1073/pnas.1800918115 10.1073/pnas.1800918115 PMC6048482 29941552

[58] Yadav P et al. Zea mays genotype influences microbial and viral rhizobiome community structure. ISME Communications, 2023;3(1):129. 10.1038/s43705-023-00335-4 10.1038/s43705-023-00335-4 PMC10700569 38057501

[59] Zhang S et al. Arbuscular mycorrhizal fungi increase grain yields: a meta-analysis’, New Phytologist. 2019;222(1): 543–555. 10.1111/nph.15570 10.1111/nph.15570 30372522

[60] Zhu X-C, Song F-B, Xu H-W. Arbuscular mycorrhizae improves low temperature stress in maize via alterations in host water status and photosynthesis. Plant and Soil. 2010;331(1):129–137. 10.1007/s11104-009-0239-z

[61] Zomer RJ et al. Climate change mitigation: A spatial analysis of global land suitability for clean development mechanism afforestation and reforestation. Agriculture, Ecosystems & Environment, 2008;126(1): 67–80. 10.1016/j.agee.2008.01.014

