# Supplementary material for: The ancestral environment of teosinte populations shapes their root microbiome
Source: Environ Microbiome. 2024 Aug 29;19:64. doi: 10.1186/s40793-024-00606-0 (PMC11363609; doi:10.1186/s40793-024-00606-0)
Supplement: Supplementary file 1 — Supplementary Material 1 [file 40793_2024_606_MOESM1_ESM.docx]

**
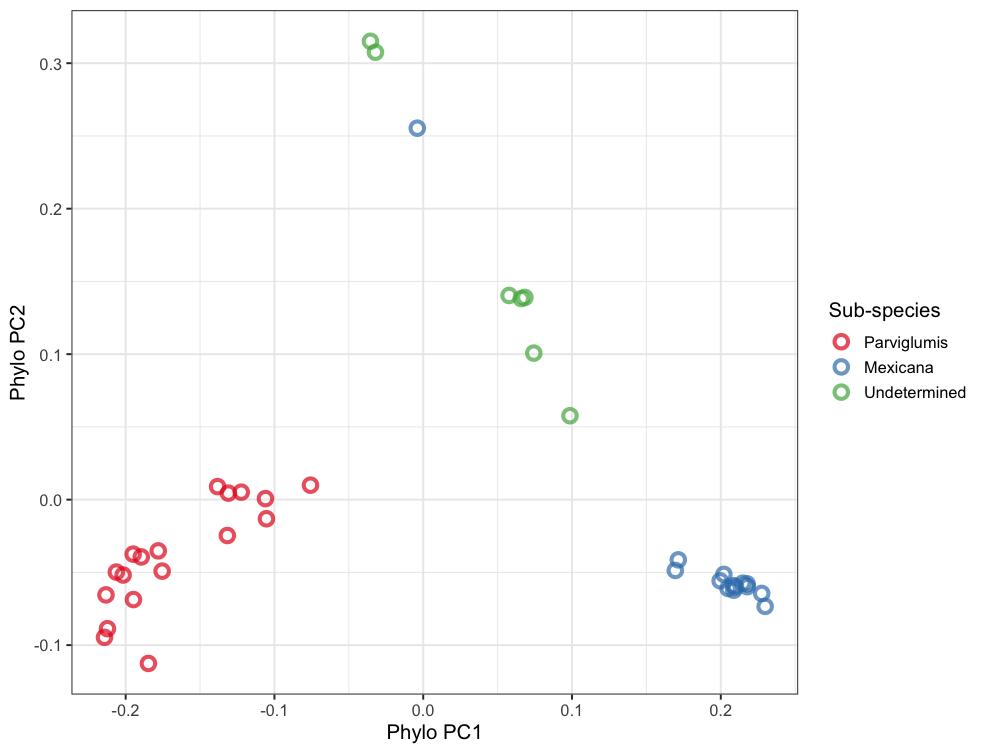
**

**Figure S1** A principal coordinate analysis was performed on the phylogenetic distance matrix, and PC1 and PC2 were plotted against each other. Samples grouped into three clusters, *Parviglumis*, *Mexicana* and likely hybrids (undetermined). One sample occurred at high elevation and was annotated as *Mexicana* using morphological analyses, and was therefore retained as a *Mexicana* sample.


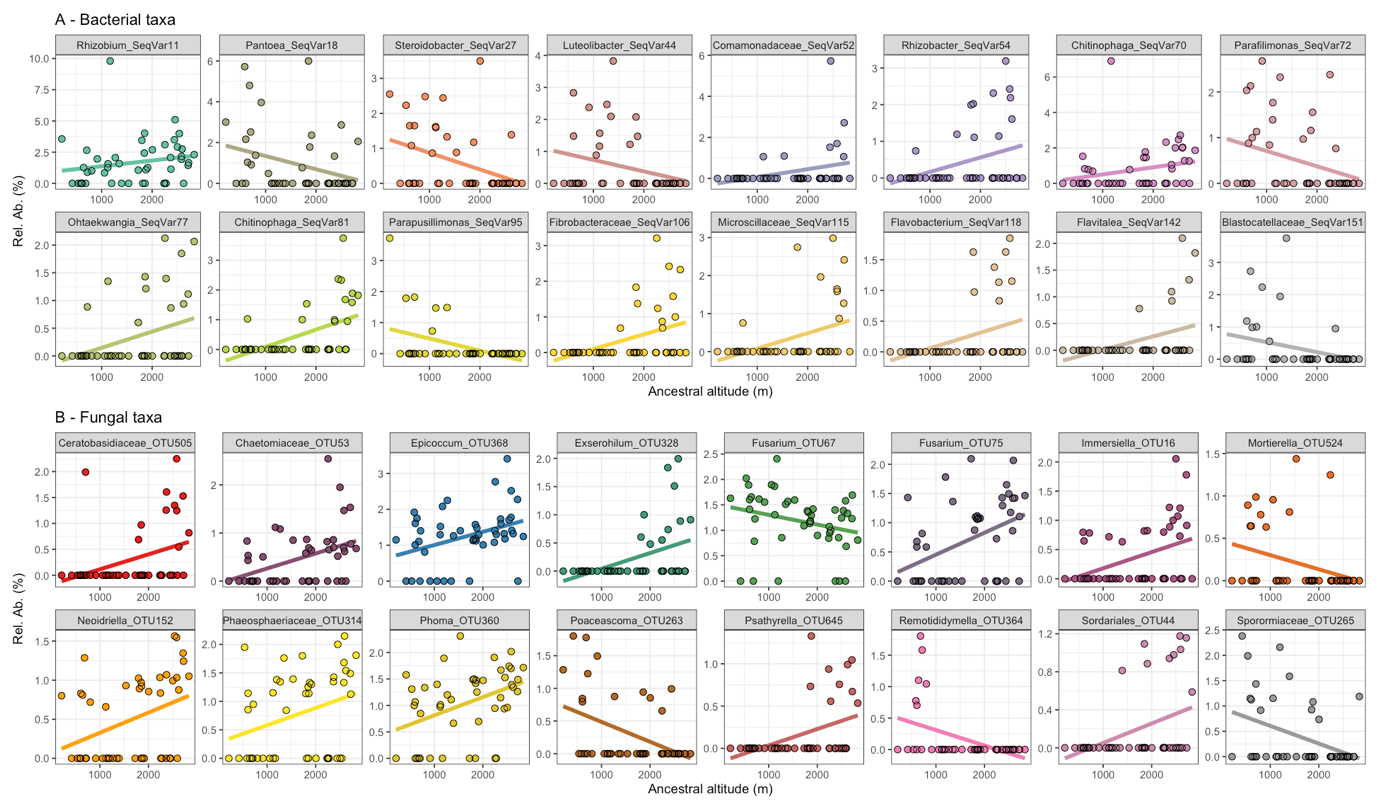


**Figure S2** The relevant abundances of abundant taxa were correlated against ancestral elevation revealing many potential correlations, while a *Chitinophaga ginsengisoli* (ASV81) also positively correlated after adjusting for multiple comparisons. Note for visualisation purposes only, untransformed ancestral elevation was plotted (by z-normalised relative abundance was used within statistics).

**
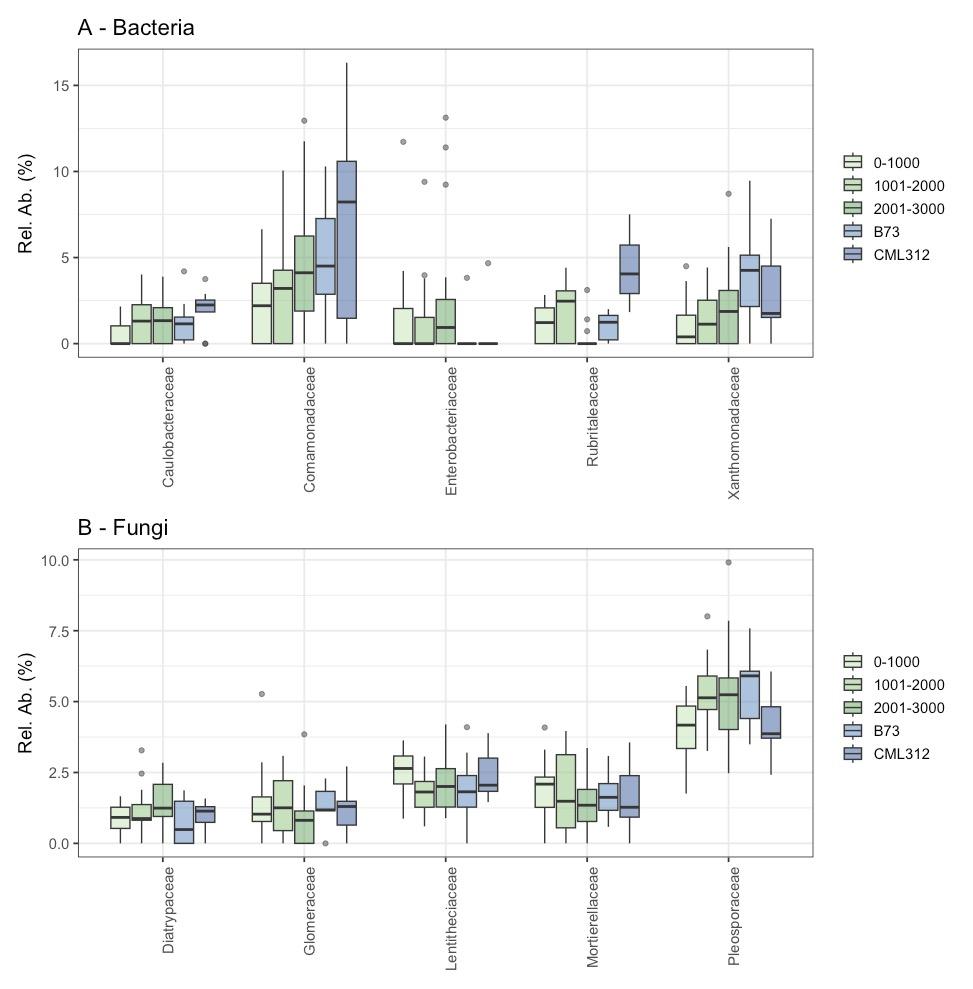
**

**Figure S3** The families that best correlated with elevation within the teosinte accessions plots were compared to the modern maize samples. Teosinte accessions were binned into low (0-1000 m), medium (1001-2000 m) and high elevations (2001-3000 m). Bars represent the lower and upper quartiles and the line represents the median. Whiskers represent the end of observed data points, with the exception of the dots, which represent outliers (defined as greater than 1.5 times the inter quarter range).
